# The PASCAL Transcatheter Valve Repair System: A User’s Guide

**DOI:** 10.1016/j.shj.2023.100204

**Published:** 2023-07-05

**Authors:** Brian Whisenant, Firas Zahr

**Affiliations:** a Intermountain Heart Institute, Structural Heart Disease, Salt Lake City, Utah, USA; b Interventional Cardiology, Oregon Health & Science University, Portland, Oregon, USA

**Keywords:** Clasp, Mitraclip, Mitral regurgitation, PASCAL, Transcatheter edge to edge repair

## Introduction

The PASCAL transcatheter valve repair system (Edwards Lifesciences, Irvine, California) is designed for percutaneous treatment of regurgitant mitral valves with reduced leaflet stress while minimizing transvalvular gradients through leaflet approximation with contoured paddles around an anatomic spacer. The device can be elongated, enabling easy retrieval from the left ventricle with reduced risk of chordal entanglement. The prospective, multinational, multicenter CLASP study^1^^,^^2^ led to CE mark approval for the treatment of primary and secondary mitral regurgitation (MR) with the PASCAL repair system, while the CLASP IID trial^3^ led to US Food and Drug Administration approval for the treatment of primary MR with the PASCAL repair system. The ongoing CLASP IIF trial is designed to establish the safety and effectiveness of the PASCAL repair system vs. MitraClip (Abbott Laboratories, Abbott Park, Illinois) for patients with secondary MR.

## Device Overview

**Figure 1:**
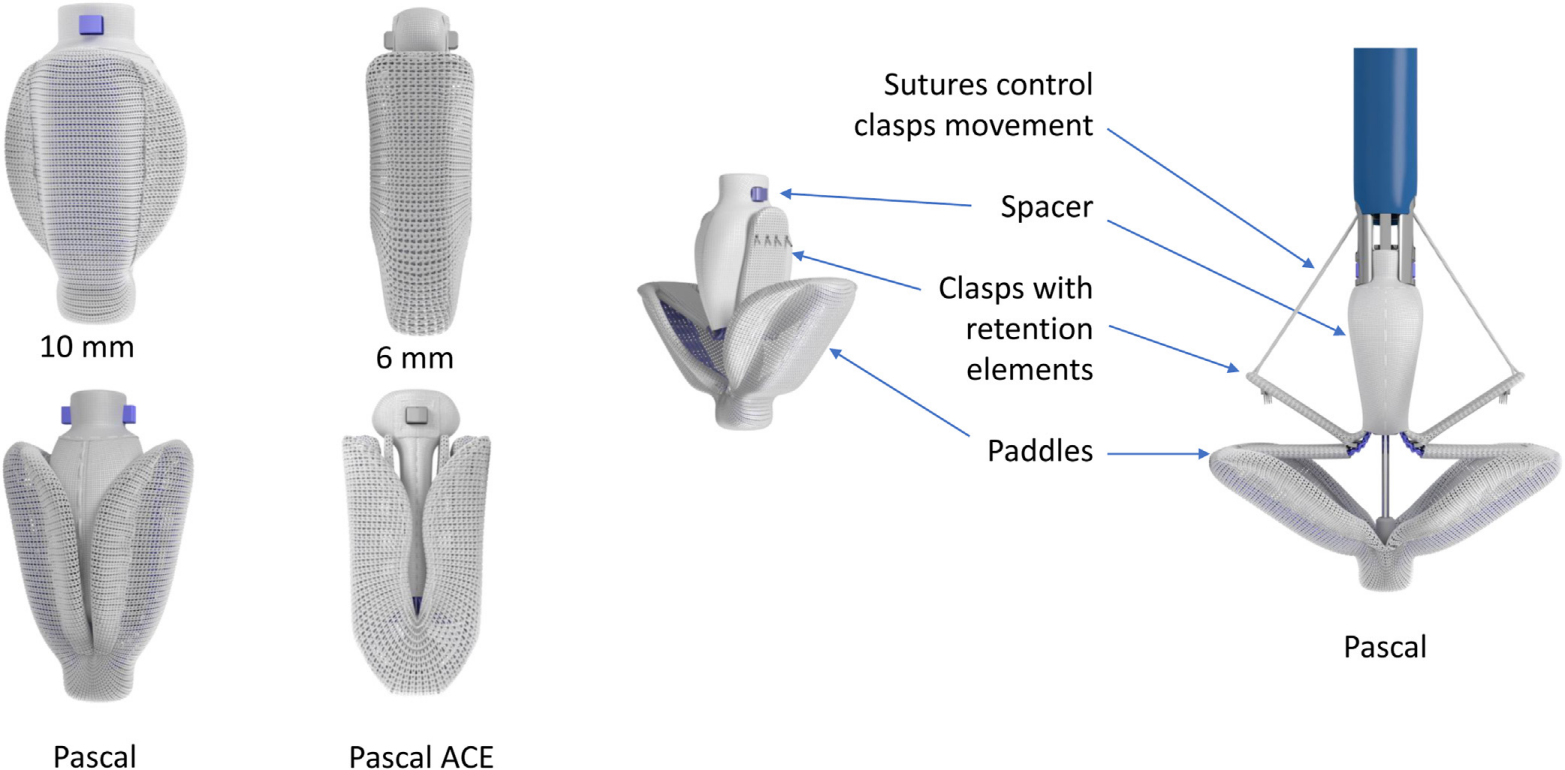
**PASCAL and PASCAL Ace implants**.

The PASCAL repair system offers 2 implant configurations; the standard PASCAL implant and the narrower PASCAL Ace implant (Figure 1). Both PASCAL implants consist of a central spacer, 2 broad and curved paddles designed to distribute force across the surface of the mitral leaflet and suture-controlled clasps capable of capturing the leaflets simultaneously or independently. The spacer provides a platform for leaflet coaptation and is designed to minimize leaflet stress and preserve mitral valve area.

The PASCAL Ace implant is functionally similar to the PASCAL implant but features narrower paddles (6 mm wide compared to 10 mm wide on the PASCAL implant) with increased curvature to distribute leaflet stress. The PASCAL Ace implant has a narrower profile and can capture slightly more leaflet length into the neocoaptation tissue bridge around a smaller spacer that is approximately half the size of the PASCAL implant while it continues to distribute tension across a broad region of leaflet tissue.

**Figure 2:**
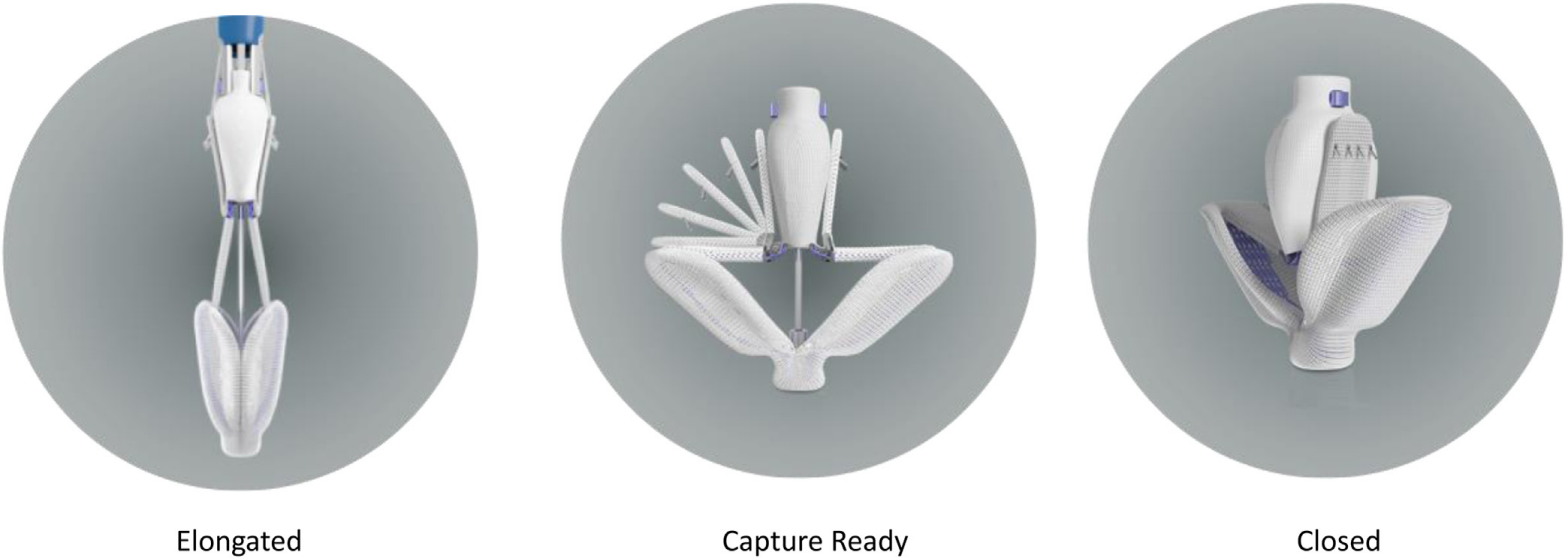
**PASCAL positions**.

Both PASCAL implants share advantageous functions. They can be fully elongated (Figure 2), a feature that helps avoid subvalvular entanglement, especially when the implant needs to be retracted from the ventricle to the atrium for repositioning. The PASCAL Ace implant is particularly well suited to regions of dense chordal structures as it allows the device to engage the leaflets with minimal interaction with the chordal structures. The clasps can be operated either simultaneously or independently to facilitate staged leaflet capture for leaflet capture optimization. The nitinol clasps have a single row of 4 retention elements near the top of the clasps distributed in a horizontal orientation. This allows engagement with the most structurally stable leaflet area for reliable retention while minimizing trauma to the tissue.

**Figure 3:**
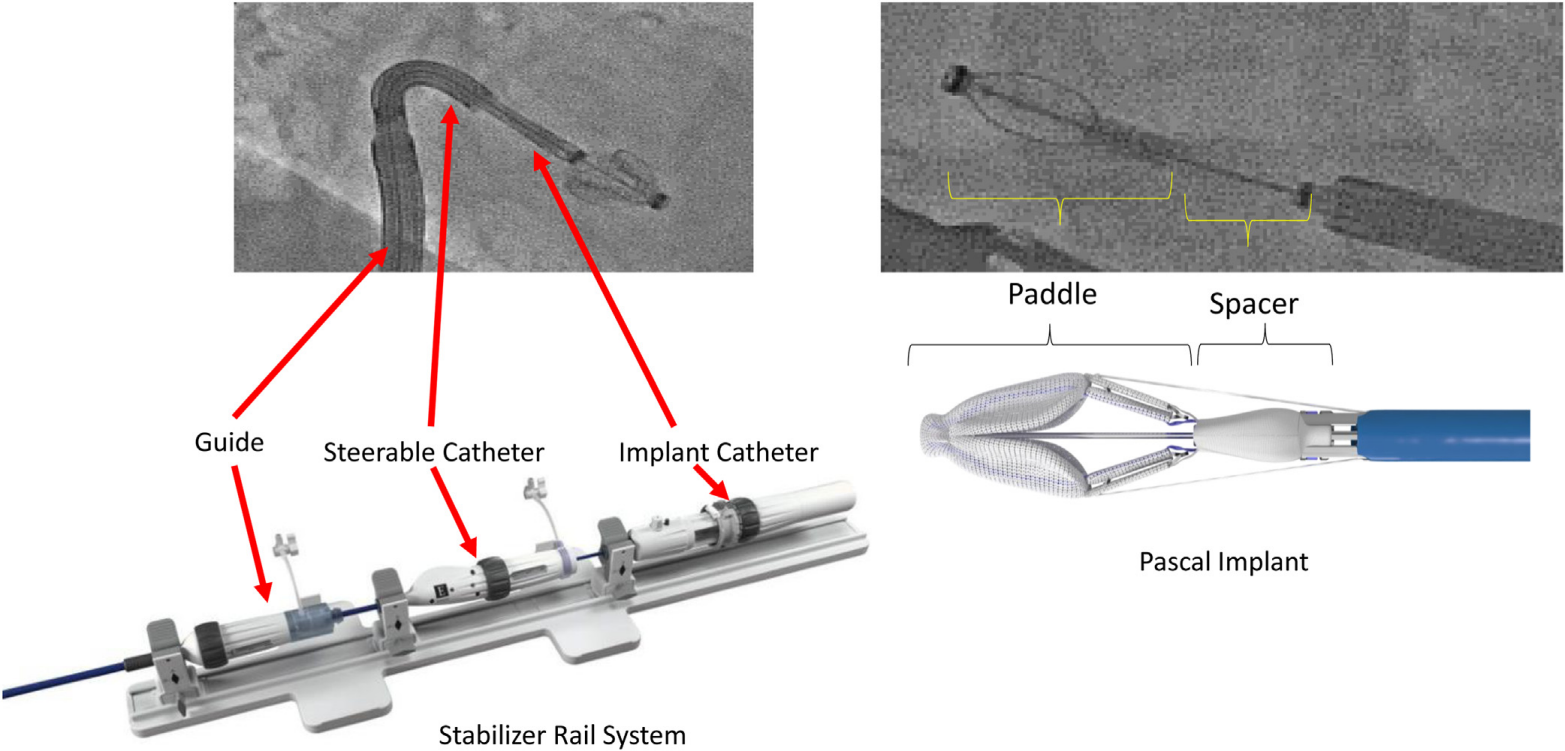
**The PASCAL transcatheter valve repair system**.

The PASCAL repair system consists of a 22 French guide sheath, a steerable catheter, and an implant catheter with the implant attached at the distal end (Figure 3). Rotational knobs on the delivery system flex the guide sheath and steerable catheter. The handle of the implant catheter controls the orientation of the implant paddles and consists of the primary controls for the implant, including the sliders that enable the clasps to be lowered simultaneously or independently, and the paddle knob which controls the implant configurations (closed, capture ready, and elongated). The implant release controls are concealed beneath the implant release cover on the proximal end of the implant catheter handle.

The Stabilizer Rail System is a platform to hold, advance, retract, and torque the catheters. The stabilizers allow for fine adjustments of the delivery system on a continuous rail, which simplifies the implant procedure while reducing unintended catheter movements throughout the procedure.

## Patient Selection

The PASCAL and PASCAL Ace implants are suitable for a broad range of anatomies of primary and secondary MR, while simultaneously addressing complex anatomies that may be difficult to treat with competing technologies. The pivotal CLASP IID trial randomized patients with anatomies suitable to the MitraClip to receive either the PASCAL repair system or the MitraClip system in a 2:1 fashion and demonstrated noninferior safety and effectiveness when compared to MitraClip. The proportion of patients with MR ≤ 1+ was durable in the PASCAL group from discharge to 6 months (PASCAL: 87.2% and 83.7% [*p* = 0.317 vs. discharge]; MitraClip: 88.5% and 71.2% [*p* = 0.003 vs. discharge], respectively). The CLASP IID registry included patients with anatomies not indicated for transcatheter edge to edge repair with MitraClip including those with ≥2 independent significant jets, a mitral valve orifice area <4.0 cm^2^, multiscallop prolapsing segments, commissural jets, and flail width >15 mm and/or a flail gap >10 mm. Despite this challenging anatomy, MR at 6 months was reduced to 1+ or less in 56% of patients and 2+ or less in 92% of treated patients.^4^ A Post Market Clinical Follow-Up Study, MiCLASP, will assess the safety and effectiveness of the PASCAL repair system and patients' improvements in functional capacity and quality of life.

Like the MitraClip system, the PASCAL repair system should be avoided in the presence of moderate to severe calcification in the grasping area, the presence of significant leaflet clefts or perforation in the grasping area, and leaflet mobility length <8 mm.

## Step-By-Step Technique

A case of PASCAL primary MR repair is demonstrated in the online Supplemental Videos 1-6.

### Access and Device Introduction

Transseptal puncture is done under transesophageal echocardiography (TEE) guidance, aiming for a mid and posterior position in the fossa ovalis at least 4.5 cm above the line of mitral leaflet coaptation. After positioning the guide sheath tip in the left atrium approximately 2 cm beyond the interatrial septum, the dilator and wire are removed. The guide sheath should not be aspirated or flushed until the implant system is inserted beyond the hemostatic seals.

After inserting the implant system with the loader into the guide sheath and advancing the implant system until it exits the loader, the loader is retracted and peeled away. The guide sheath is then aspirated of 45 cc of blood and flushed with heparinized saline. A pressure line can be connected to the steerable catheter for continuous left atrial pressure monitoring.

**Figure 4:**
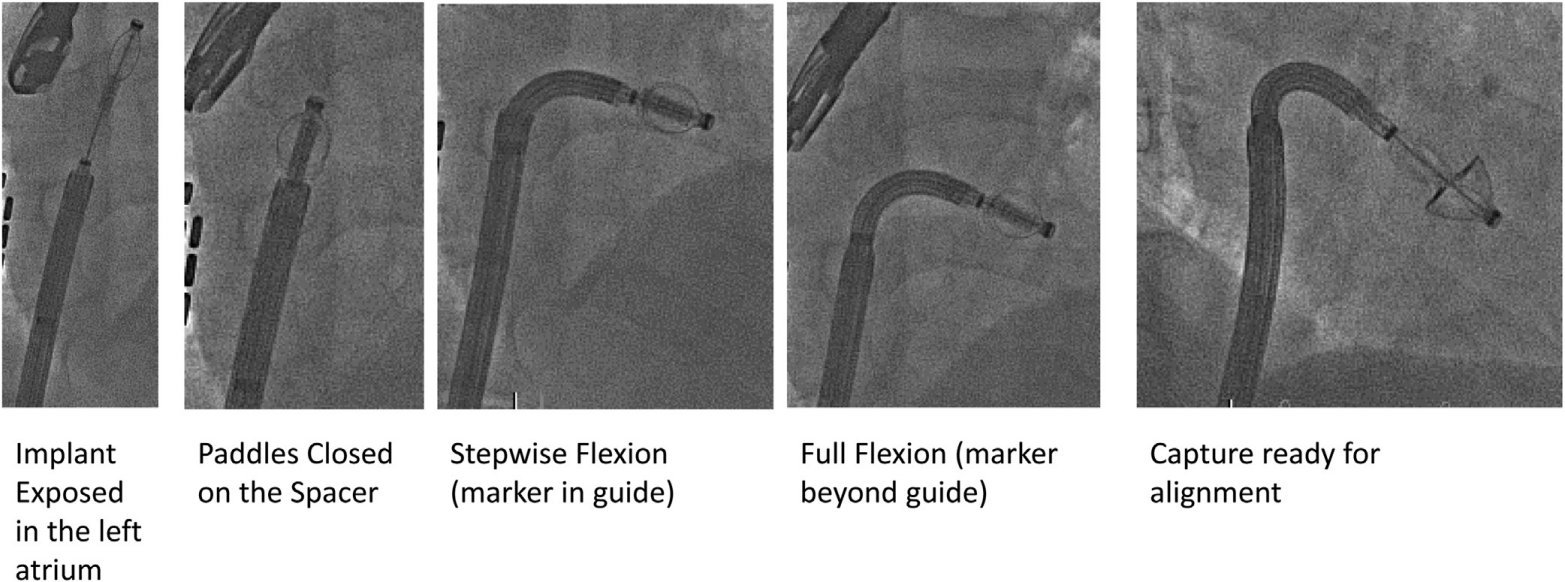
**PASCAL introduction**.

The elongated implant is introduced into the left atrium until at least the paddle spacer junction is visualized beyond the guide catheter tip on fluoro (Figure 4). Ideally, the full implant is exposed in the left atrium allowing the paddles to be closed on the spacer. If space in the left atrium is limited, the paddles may be closed on the flexible portion of the guide sheath tip and then further advanced to expose the spacer.

### Device Navigation

Prior to flexing the delivery system to the mitral valve, it is helpful to orient the system with the guide sheath’s flush port at the 4 o’clock position, the steerable catheter’s fin at the 2 o’clock position, and the implant catheter flush port at the 12 o’clock position.

The steerable catheter is flexed toward the mitral valve under TEE guidance in a stepwise fashion beginning as the steerable catheter exits the guide sheath. The marker band on the steerable catheter indicates the end of the flex section and is used as a fluoroscopic visual aid to confirm the full flex region has exited the tip of the guide sheath. The PASCAL implant is positioned over the targeted leaflet segment and finely adjusted to ensure a coaxial approach. While the steerable catheter is primarily used to optimize the implant position and trajectory, the guide sheath can be flexed to optimize the trajectory when experiencing “aorta huggers” and used to gain height if the transeptal puncture is too low or there is a need to be higher in the atrium (i.e., significant flail or prolapse of the leaflet into the atrium).

Flexing the guide sheath moves the distal portion of the system posteriorly away from the aorta and medial, helping to achieve a more perpendicular trajectory to the valve. Steerable catheter retraction and advancement helps position the implant in the medial to lateral direction. Steerable catheter flex and unflex changes the implant trajectory in the medial to lateral direction. The steerable catheter moves independently of the guide sheath to achieve an optimal implant position and trajectory. If needed, additional height above the mitral annulus can be achieved via posterior rotation of the guide sheath, countered with anterior rotation of the steerable catheter to maintain an optimal position over target pathology.

### Leaflet Capture

Once the PASCAL implant is centered over the target pathology with an optimal trajectory, the paddles are opened to the capture-ready position (Images 2, 4, and Supplemental Video 2). The clasps are identified and tested individually, and the implant is rotated under 3D TEE (ideally multiplane reconstruction) by rotating the implant catheter handle to orient the paddles perpendicular to the line of mitral leaflet coaptation. Rotation of the implant catheter to achieve implant alignment may cause modest secondary anterior or posterior deviation of the delivery system. This is countered by opposite rotation of the steerable catheter handle to keep the implant centered over the targeted pathology.

The implant has 3 main configurations: elongated, capture-ready, and closed (Figure 2), which are controlled by rotating the paddle knob on the implant catheter. The valve is traditionally crossed in the capture-ready position with the paddles open to 180° under TEE guidance to maintain an orientation perpendicular to the line of coaptation (Supplemental Videos 2 and 3). The implant catheter is retracted until the mitral leaflets are visualized by TEE with full leaflet insertion onto the paddles (Supplemental Video 3).

The leaflets may be grasped either simultaneously or independently. Full leaflet insertion is essential (Supplemental Videos 3 and 4). Leaflet optimization is commonly performed by first opening the implant to capture ready, rotating the steerable catheter to bias the implant anteriorly or posteriorly toward the targeted leaflet, retracting the implant catheter slightly, raising the target clasp to allow for deeper leaflet engagement, and then again lowering the clasp and closing the device. Optimization can be performed for either the anterior or posterior leaflet or for both simultaneously.

When full (9 to 10 mm) leaflet insertion is confirmed, the device is closed until the paddle knob clicks. Residual MR and transvalvular gradient are systematically assessed before final deployment. The implant can be optimized, repositioned, or removed, if required.

### Release

When an optimal result is confirmed, the implant is fully deployed and released by removing the implant release cover to expose the suture locks and implant release knob. Unscrew each suture lock and remove the sutures. After suture removal, the blue implant release knob is rotated counterclockwise and simultaneously retracted until the implant is released and seen to separate from the catheter on fluoroscopy.

If necessary, and if the residual orifice area is acceptable, a second device may be implanted. Rather than crossing the mitral valve in a capture-ready position, a second device can be advanced in the elongated or the closed position, and then configured to capture-ready below the mitral leaflets.

## Procedural Pearls

• Orientation of the paddles through rotation of the implant catheter is often associated with anterior or posterior deflection of the implant system requiring a slight counter rotation of the steerable catheter.
• The distributive advantages of broad paddles are well understood. However, broader paddles may also encounter chords, which may prevent full leaflet insertion or redirect the device. The subvalvular apparatus guides the PASCAL implant position and orientation. When chords are encountered, the paddles may be moved medial or lateral or the implant may be slightly rotated. Opening and closing the paddles between elongated, capture-ready, and fully closed may allow the implant to work its way between chords. In certain circumstances, the narrower PASCAL Ace implant may be able to navigate more easily through chords and avoid chordal interference.
• The retention force between the paddles and clasps is constant between capture-ready and the fully closed position. The capture ready position provides the broadest orientation for leaflet engagement and a stable configuration for TEE interrogation of leaflet insertion. Releasing the implant prior to confirmation that each leaflet is fully and securely captured between paddle and clasp may result in single leaflet device attachment. After the implant is closed, poor leaflet visualization and MR reduction can provide false confidence of adequate leaflet insertion. As the depth of leaflet insertion is best visualized in the capture-ready position, time should be taken to confirm full leaflet insertion with TEE after the clasps are lowered and before the device is closed. Systolic motion of the clasps suggests leaflet engagement but may be insufficient to ensure a minimum of 6 mm of leaflet insertion for optimal retention. Asymmetric paddle flex visualized on fluoro after device closure suggests excessive leaflet or possibly chords captured between the paddles and clasps.
• The orientation of the first implanted PASCAL defines the coaptation plane and thereby dictates the orientation of subsequently implanted devices. Malorientation can lead to suboptimal MR reduction. As both the PASCAL and PASCAL Ace implants capture a broad segment of leaflet tissue, excellent MR reduction can often be achieved with a single device carefully positioned and oriented for optimal MR reduction.

## Clinical Evidence

The first patient was treated with the PASCAL repair system in Switzerland in 2016. CE mark approval of the PASCAL repair system was obtained in 2019 based on the data from the multinational, multicenter, prospective CLASP study. Sixty-two patients with primary (degenerative MR) or secondary (functional MR) MR were included at 14 sites. The rate of MR grade ≤2 + 30 days after the procedure was 98%, and all-cause mortality was 1.6%. No leaflet damage and only 1 single leaflet device attachment were observed. The mean gradient was 4.1 ± 1.8 mmHg after implantation of 1.5 implants/patient and remained stable during follow-up. At 6 months, 98% of the patients had MR grade ≤2+ and 81% had MR grade ≤1+, suggesting a stable MR reduction over time. Two-year data show 97% of patients had MR grade ≤2 + and 87% had MR ≤ 1 +, indicating durable MR reduction. Furthermore, evidence of left ventricular reverse remodeling was demonstrated.”^3^^,^^5^^,^^6^

The PASCAL CLASP IID/IIF clinical trial (NCT03706833) is a prospective, multicenter, randomized, controlled pivotal trial designed to compare the safety and effectiveness of transcatheter edge to edge repair with the PASCAL Transcatheter Valve Repair System to MitraClip in patients with degenerative MR who have been determined to be at prohibitive risk for mitral valve surgery by the heart team, and in patients with functional MR on guideline directed medical therapy. The CLASP IID/IIF trial is the first head-to-head study comparing 2 transcatheter mitral valve repair devices and is designed as a noninferiority trial. The CLASP IID trial demonstrated noninferiority of PASCAL compared with MitraClip at 6 months; 1-year follow up is underway. The CLASP IIF trial is underway and is randomizing patients with secondary MR to PASCAL repair system or MitraClip system.

## Summary

The PASCAL transcatheter valve repair system, with the PASCAL and PASCAL Ace implants, is a novel treatment option with design features that are well suited for a broad range of straightforward and complex anatomies. Growing commercial and investigational experience support the safety, efficacy, and durability of the PASCAL repair system for transcatheter mitral valve leaflet repair. With the recent completion of the CLASP IID trial along with US Food and Drug Administration approval, PASCAL is now an essential treatment option for MR.

## Consent Statement

Individual informed consent was not obtained for this specific publication.

## Funding

The authors have no funding to report.

## Disclosure Statement

Brian Whisenant reports a relationship with Edwards Lifesciences Corporation that includes: consulting or advisory and speaking and lecture fees. Brian Whisenant reports a relationship with Abbott Cardiovascular Structural Heart Division that includes: consulting or advisory.

## Supplementary Material

Supplemental Videos 1-6
